# Efficacy and safety of selected Chinese herbal medicine preparations in treating carotid atherosclerosis: a systematic review and meta-analyses

**DOI:** 10.3389/fphar.2026.1903096

**Published:** 2026-10-02

**Authors:** Yuemeng Sun, Menghan Feng, Haoran Zhang, Jiayi Liu, Qian Li, Shujing Zhang, Chunjie Ma, Hao Wu

**Affiliations:** 1 College of Traditional Chinese Medicine, Inner Mongolia Medical University, Hohhot, China; 2 School of Medicine, Hetao College, Bayannur, China; 3 School of Traditional Chinese Medicine, Beijing University of Chinese Medicine, Beijing, China

**Keywords:** carotid atherosclerosis, Chinese herbal medicine, medication patterns, meta-analysis, systematic review

## Abstract

**Background:**

Although conventional therapies for Carotid atherosclerosis (CAS) remain the standard treatment, their effectiveness is often limited and accompanied by adverse effects. Chinese herbal medicine (CHM) is a promising adjunctive therapy for CAS; however, robust clinical evidence supporting its efficacy and safety remains limited.

**Objective:**

This systematic review and meta-analysis evaluates the efficacy and safety of CHM for CAS treatment and characterizes its medication patterns.

**Methods:**

Five databases were searched from inception to December 2025. Randomized controlled trials (RCTs) evaluating oral CHM for CAS were included. Study quality was assessed using the Cochrane Risk of Bias 2 (RoB 2) tool. Data were extracted and analyzed using RevMan v5.3 and R software. Sources of heterogeneity were investigated through meta-regression and subgroup analyses. Network analysis reveals herbal medication patterns across 21 studies.

**Results:**

Twenty-one RCTs involving 3,078 patients were included. Compared with conventional therapy alone, adjunctive CHM significantly reduced carotid plaque area (MD = 4.94, 95% CI: 3.13 to 6.76, *p* < 0.01), intima-media thickness (IMT) (MD = 0.12, 95% CI: 0.07 to 0.17, *p* < 0.05), total cholesterol (TC) (MD = 0.32, 95% CI: 0.22 to 0.42, *p* < 0.01), triglycerides (TG) (MD = 0.24, 95% CI: 0.11 to 0.37, p < 0.01), and low-density lipoprotein cholesterol (LDL-C) (MD = 0.37, 95% CI: 0.21 to 0.53, *p* < 0.01), while greatly increasing high-density lipoprotein cholesterol (HDL-C) (MD = −0.20, 95% CI: −0.29 to −0.10, *p* < 0.01). CHM did not significantly increase the risk of adverse events (RR = 0.84, 95% CI: 0.64 to 1.10, *p* = 0.20) or impair liver/kidney function. Network analysis identified core actions as clearing phlegm and removing blood stasis, with Danshen (Salvia miltiorrhiza Bge.) and Banxia [Pinellia ternata (Thunb.) Breit.] as pivotal botanical drugs exerting anti-stasis and lipid-regulating effects.

**Conclusion:**

As an adjunct to conventional therapy, CHM may further reduce carotid IMT and plaque burden, improve lipid metabolism, and demonstrate a favorable safety profile. However, the overall quality and heterogeneity of the included studies limit the strength of the evidence, highlighting the need for well-designed, high-quality RCTs to confirm these findings.

**Systematic Review Registration:**

https://www.crd.york.ac.uk/PROSPERO/view/CRD420261285381, identifier CRD420261285381.

## 1 Introduction

Carotid atherosclerosis (CAS) is a major contributor to ischemic stroke and cardiovascular disease (Fu et al., 2024; Parish et al., 2019; Hald et al., 2014; Li et al., 2022a). It most commonly develops at the carotid bifurcation and within the distal common carotid and proximal internal carotid arteries. The pathogenesis of CAS is closely linked to lipid metabolism abnormalities and changes in local hemodynamic forces (Zhang et al., 2022; Li et al., 2021; Motomiya and Karino, 1984). Increased intima-media thickness (IMT), carotid plaque formation, and varying degrees of carotid artery stenosis are recognized as key indicators of disease progression (Nai et al., 2025; Guo et al., 2025; Almevall et al., 2025). Epidemiological studies estimate that the global prevalence of CAS among adults reaches approximately 50.25% (Song et al., 2020), with similarly high prevalence reported in China (Fu et al., 2024). Current conventional management of CAS primarily focuses on lipid-lowering and antiplatelet therapies, both of which have demonstrated clinical benefit (Gresele et al., 2020; Hu et al., 2022a). Statins remain first-line agents for lipid reduction (Jia et al., 2021; Kadoglou et al., 2022; Huang et al., 2025); however, they may cause adverse effects, including myalgia and, in rare cases, rhabdomyolysis (Adhyaru et al., 2018; Thompson et al., 2016; Sizar et al., 2024). Antiplatelet therapy, particularly aspirin, is widely used for cardiovascular prevention, but its benefit in asymptomatic CAS remains limited, and prolonged treatment is associated with an increased risk of bleeding (Gresele et al., 2020; Hu et al., 2022a).

Chinese herbal medicine (CHM) uses a multi-component, multi-target, multi-pathway therapeutic strategy. Growing evidence suggests that CHM may improve lipid metabolism by reducing low-density lipoprotein cholesterol (LDL-C), triglycerides (TG), and total cholesterol (TC) (Jiang et al., 2025; Ma and Zhang, 2025; Yuan et al., 2025). Clinical studies have also reported favorable effects of herbal therapies on lipid profiles, clinical symptoms, and laboratory indicators in patients with CAS (Chang et al., 2025; Sun et al., 2025).

To provide a more comprehensive assessment of the available evidence, this study systematically reviewed and synthesized data from published randomized controlled trials (RCTs) investigating CHM for CAS. The primary objective was to evaluate its clinical efficacy and safety. Additionally, we employed network topology to explore the medication patterns of CHM for CAS, aiming to provide preliminary evidence to support more rational and optimized clinical application.

## 2 Study methodology

This study was conducted in accordance with the *Preferred Reporting Items for Systematic Review and Meta-Analysis Protocols* (PRISMA-P) guidelines.

### 2.1 Registration

The study is registered in the International Prospective Register of Systematic Reviews (PROSPERO; Registration No.: CRD420261285381).

### 2.2 Inclusion criteria

- Design of included studies: Randomized controlled trials (RCTs) only.
- Participants: Studies enrolling participants with a confirmed diagnosis of CAS were considered eligible. CAS was diagnosed according to the following criteria: carotid IMT thickening was defined as an IMT ≥1.0 mm, whereas carotid plaque was defined as an IMT >1.5 mm, a focal increase in IMT of at least 0.5 mm compared with the adjacent normal arterial wall, or a focal thickening exceeding 50% of the surrounding IMT accompanied by protrusion into the arterial lumen (Touboul et al., 2012; Cerebrovascular Disease Society and Chinese Society of Neurology, 2017; Chinese Society of Health Management et al., 2015). No restrictions were applied regarding participants’ age, sex, disease duration, or the presence of comorbidities.
- Interventions: The experimental group received traditional CHM either as monotherapy or in combination with the interventions administered to the control group. Botanical drug interventions included single-botanical-drug preparations, botanical drug pairs, and multi-botanical-drug compound formulations.

The control group received conventional biomedical care, a placebo, or a combination of conventional therapy and placebo.

d. Outcome Measures:

Primary outcomes: IMT, Carotid plaque area, Carotid plaque volume.

Secondary outcomes: TC, TG, LDL-C, HDL-C.

Safety outcomes: Indicators of liver and kidney function, adverse events (AE), and adverse reactions.

### 2.3 Study exclusion criteria

Research that satisfied any of the following requirements was eliminated.

- Duplicate publications.
- Inconsistent interventions, incomplete primary outcome data, or inability to contact the authors for clarification.
- Missing data or evident methodological or reporting errors.
- Received botanical drug treatment without adherence to TCM syndrome differentiation.

### 2.4 Literature search strategy

Two reviewers independently searched Embase, the Cochrane Library, Web of Science, PubMed, and the China National Knowledge Infrastructure (CNKI) from database inception through December 2025. The search strategy combined Medical Subject Headings (MeSH) with free-text keywords. Detailed search strategies for each database are presented in Table 1.

**TABLE 1:** Search strategy.

No.	Search expression
#1	“Carotid artery diseases” [MeSH terms]
#2	“Carotid atherosclerosis” or “carotid artery disease” or “carotid arterial diseases” or “carotid arterial diseases” or “carotid artery disorders” or “carotid artery disorder” or “carotid artery disorders” or “carotid artery disorders” or “carotid artery disorders” or “carotid artery disorders” or “carotid artery diseases” or “internal carotid arterial diseases” or “carotid atherosclerosis” or “carotid atherosclerotic disease”
#3	#1 OR #2
#4	“Chinese herbal drugs” [MeSH terms]
#5	“Chinese medicinal plants” or “Chinese herbal medicines” or “herbal medicines (Chinese)” or “Chinese plant extracts” or “Chinese medicinal plant extracts” or “extracts from Chinese medicinal plants”
#6	#4 OR #5
#7	#3 AND #6

Following initial screening, the reference lists of all included studies and relevant review articles were manually searched to identify additional eligible studies.

### 2.5 Literature selection and data extraction

Two reviewers conducted study selection independently, and cross-checked the results to ensure consistency. Reviewers resolved disagreements through discussion and consensus or, when necessary, by consulting subject-matter experts.

The screening process followed predefined eligibility criteria. Studies that did not meet the inclusion criteria were excluded, including irrelevant publications and retracted studies. Then, titles and abstracts were screened, and only RCTs evaluating CHM for the treatment of CAS were retained. Full-text articles were then assessed to remove duplicate publications and studies lacking essential data when the corresponding authors could not be contacted.

Standardized data were extracted independently from the included studies using Microsoft Excel. The extracted information comprised: (a) general study characteristics, including the title, first author, and publication year; (b) baseline participant characteristics, including sample size, sex distribution, age, and disease duration; (c) details of the intervention protocols; (d) information relevant to risk-of-bias assessment; (e) predefined outcome measures of interest.

To explore the medication patterns of CHM for CAS, bipartite networks were constructed linking the compound formulas, their constituent botanical drugs, and the pharmacological actions reported in the included studies.

### 2.6 Risk-of-bias assessments

Two reviewers independently assessed the methodological quality of the included studies using the revised Cochrane Risk of Bias 2 (RoB 2) tool (Sterne et al., 2019), and cross-checked the results for consistency. The assessment covered seven domains: bias arising from the randomization process; bias due to deviations from intended interventions; bias due to missing outcome data; bias in measurement of the outcome; bias in selection of the reported result. Each domain and the overall risk of bias are rated as low risk, some concerns, or high risk.

### 2.7 Statistical analysis

Statistical analyses were performed using RevMan v5.3 and R software. Dichotomous outcomes were summarized as relative risks (RRs). Continuous outcomes were expressed as mean differences (MDs) when measurement units were consistent across studies or as standardized mean differences (SMDs) when different measurement scales were used. All effect estimates were reported with corresponding 95% confidence intervals (CIs).

Between-study heterogeneity was assessed using the Chi-square test (α = 0.10) and quantified with the I^2^ statistic. A fixed-effects model was applied when heterogeneity was low (*I* ^*2*^ ≤ 25%), whereas a random-effects model was used for moderate heterogeneity (25% < *I* ^*2*^ ≤ 75%). When substantial heterogeneity was detected (*I* ^*2*^ > 75%), potential sources were explored through appropriate analyses. When clinical heterogeneity was evident, subgroup or sensitivity analyses were conducted where feasible; otherwise, the findings were summarized descriptively.

Network visualization and topology analysis were performed using Cytoscape (version 3.10). Degree centrality was computed using the CentiScaPe plugin (version 2.2) to identify core pharmacological actions and pivotal botanical drugs.

### 2.8 Reference standards

The diagnostic criteria for CAS were based on the Mannheim Carotid Intima-Media Thickness and Plaque Consensus (2004–2006–2011) (Touboul et al., 2012) and the Chinese Consensus on the Diagnosis and Treatment of Atherosclerosis of the Head and Neck Arteries (Cerebrovascular Disease Society and Chinese Society of Neurology, 2017).

## 3 Results

### 3.1 Outcomes retrieval

The literature search identified a total of 3,366 records. After the initial screening, 141 duplicate records, four studies with unavailable full texts, and one retracted publication were excluded. Title and abstract screening further eliminated 3,056 records, including basic research articles, case reports, review papers, data analyses, and studies unrelated to CAS, leaving 164 articles for full-text assessment. Following detailed full-text review, studies were excluded because of incomplete data, non-traditional CHM interventions, or a high risk of bias. Ultimately, 21 randomized controlled trials (Gong et al., 2019; Cheng et al., 2021; Fan et al., 2024; Shen et al., 2022; Zhang et al., 2019; Xie et al., 2025; Zhao et al., 2023; Zhang et al., 2022; Wu et al., 2010; Wu et al., 2000; Wei et al., 2026; Wang et al., 2014; Shen et al., 2012; Mao et al., 2011; Liu et al., 2016; Liu et al., 2019; Liu et al., 2014; Jiang and Zhang, 2022; Chen and He, 2024; Zeng et al., 2021; Cao and Wang, 2013) met the eligibility criteria and were included in the final analysis (Figure 1).

**FIGURE 1:**
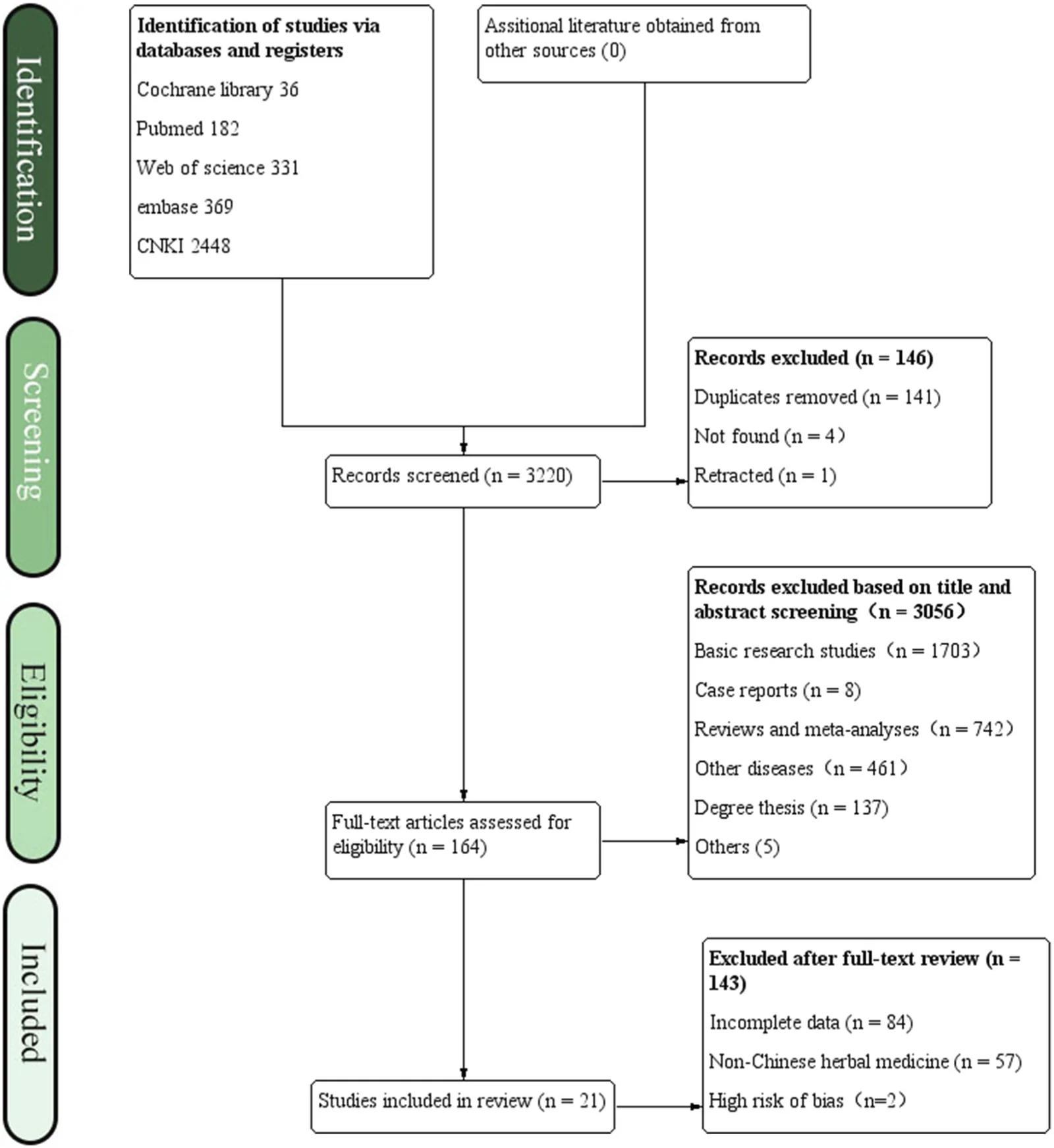
Flowchart of the literature screening procedure.

### 3.2 Study characteristics

All RCTs in this meta-analysis were conducted in China and enrolled 3,078 participants, with 1,555 assigned to the CHM group and 1,523 to the control group. Baseline demographic and clinical characteristics were comparable between the intervention and control groups across all included studies. All participants underwent carotid IMT assessment before treatment, and no statistically significant differences in baseline IMT or other measured characteristics were observed between the CHM and control groups (*p* > 0.05; Table 2).

**TABLE 2:** Study information.

S.No	Author	Journal	Number of cases		Mean age (years)	Intervention		Duration of treatment/months	Outcome
			Treatment (T)	Control (C)		Treatment (T)	Control (C)	​	​
1	Mei zhang 2019	Scientific reports	607	605	61.4 ± 8.4	Tongxinluo capsule + routine therapy	Placebo capsules + routine therapy	24	IMT, carotid artery plaque area, TC, TG, LDL-C, HDL-C, adverse events
2	Guangqing cheng 2021	Evidence-based complementary and alternative medicine	45	45	67.1 ± 7.61	Huayu tongmai formula + control	Aspirin enteric-coated tablets, atorvastatin calcium tablets	3	IMT, TC, TG, ldl-c, HDL-C
3	Xu shen 2022	Phytomedicine	72	71	64.4 ± 7.46	Dengzhan shengmai capsule	Aspirin enteric-coated tablets	12	IMT, TC, TG, LDL-C, HDL-C, adverse events, liver and kidney function
4	Jianhua fan 2024	Medicine	60	60	57.2 ± 15.33	Jiangzhi maian granules + control	Aspirin enteric-coated tablets, atorvastatin calcium tablets, isosorbide mononitrate sustained-release tablets	3	IMT, carotid artery plaque area, TC, TG, LDL-C, HDL-C, liver and kidney function
5	Baoying gong 2019	Evidence-based complementary and alternative medicine	59	59	64.8 ± 7.079	C-117 + control	Placebo	6	IMT, carotid artery plaque area, TC, TG, LDL-C, HDL-C, liver and kidney function, adverse events
6	Jingbi xie 2025	Chinese journal of clinical rational drug use	40	39	46.7 ± 6.25	Modified banxia baizhu tianma decoction + control	Atorvastatin calcium tablets Amlodipine besylate tablets	2	IMT, carotid artery plaque area, TC, TG, LDL-C, HDL-C, adverse events
7	Ruonan zhao 2023	Lishizhen medicine and materia medica research	30	30	63.9 ± 9.16	Xiaorong wenban granules + control	Aspirin enteric-coated tablets, atorvastatin calcium tablets	6	IMT, carotid artery plaque area, TC, TG, LDL-C, HDL-C, adverse events
8	Youhua zhang 2013	Proceedings of the 3rd forum on scientific and technological achievements in traditional Chinese medicine	120	100	64.0 ± 4.50	Xiaoban tongmai formula	Simvastatin capsules, clopidogrel tablets, aspirin	3	IMT, carotid artery plaque area, TC, TG, LDL-C, HDL-C, adverse events
9	Tianmin Wu 2010	Guangming journal of Chinese medicine	30	30	59.5 ± 7.8	Kangxin capsule	Atorvastatin calcium tablets	3	IMT, carotid artery plaque area, TC, TG, LDL-C, HDL-C, adverse events
10	Shengxian Wu 2000	Fujian journal of traditional Chinese medicine	17	15	56.6 ± 7.60	Jiedu ruanmai formula	Aspirin enteric-coated tablets	3	IMT, carotid artery plaque area, TC, TG, LDL-C, HDL-C
11	Yulian wei 2024	Jiangsu journal of traditional Chinese medicine	30	30	67.2 ± 9.20	Xiaozhi huaban formula + control	Atorvastatin calcium tablets	3	IMT, TC, TG, LDL-C, HDL-C, adverse events
12	Peijun wang 2014	Medical innovation of China	24	24	31.5 ± 4.91	Gegen qinlian decoction + control	Simvastatin	6	IMT, TC, TG, ldl-c, HDL-C
13	Dingzhu shen 2012	Journal of emergency in traditional Chinese medicine	40	40	58.61 ± 4.15 ​	Shoushen granules + control	Pravastatin	6	IMT, TC, TG, LDL-C, HDL-C, adverse events
14	Junmin mao 2011	Modern journal of integrated traditional Chinese and western medicine	50	45	54.46 ± 7.25	Jiangzhi xiaoji yin	Simvastatin	6	IMT, TC, TG, LDL-C, HDL-C, adverse events
15	Dan Liu 2016	Journal of sichuan traditional Chinese medicine	40	40	58.7 ± 6.3	Jianpi quyu formula	Simvastatin	6	IMT, TC, TG, LDL-C, HDL-C, adverse events
16	Chuanliang Liu 2019	World Latest medicine information	92	92	64.20 ± 4.84	Compound danshen chuanxiong dispensing granules + control	Aspirin, atorvastatin calcium	3	IMT, carotid artery plaque area, TC, TG, LDL-C, HDL-C, adverse events
17	Aijun Liu 2014	Journal of sichuan traditional Chinese medicine	39	39	63.75	Huazhuo tongluo decoction + control	Antihypertensive drugs, antiplatelet agents, statins	6	IMT, carotid artery plaque area, TC, TG, LDL-C, HDL-C
18	Xiaojun jiang 2022	Shanxi journal of traditional Chinese medicine	30	30	62.47 ± 5.95	Wenyang tongmai Formula + control	Atorvastatin calcium tablets	9	IMT, carotid artery plaque area, TC, TG, LDL-C, HDL-C, adverse events
19	Xiuwen chen 2024	Guangming journal of Chinese medicine	30	29	61.77 ± 8.37	Qingxiao tongyi formula + control	Aspirin enteric-coated tablets, atorvastatin calcium tablets, clopidogrel tablets	12	IMT, carotid artery plaque area, TC, TG, LDL-C, HDL-C
20	Jing zeng 2021	Modern journal of integrated traditional Chinese and western medicine	40	40	54.4 ± 5.0	Shugan wendan decoction + control	Atorvastatin calcium tablets	3	IMT, carotid artery plaque area, TC, TG, LDL-C, HDL-C, adverse events
21	Xiaoling cao 2013	Journal of sichuan traditional Chinese medicine	60	60	65.55	Yiqi huatan huoxue formula	Aspirin enteric-coated tablets, rosuvastatin tablets	6	IMT, carotid artery plaque area, TC, TG, LDL-C, HDL-C, adverse events

Abbreviations: T, treatment group; C, control group; IMT, intima-media thickness; TC, total cholesterol; TG, triglycerides; LDL-C, low-density lipoprotein cholesterol; HDL-C, high-density lipoprotein cholesterol.

All included studies received approval from their respective institutional ethics committees and were conducted in accordance with Good Clinical Practice guidelines. The botanical drug formulations used were all included in the Pharmacopeia of the People’s Republic of China, and the prescribed dosages were within the Pharmacopeia-specified therapeutic ranges, ensuring compliance with established standards for quality, safety, and clinical application. Supplementary Table S1 present detailed characteristics of the included studies and botanical drug interventions.

### 3.3 Quality assessment of literature

Several studies, including those by Cheng et al., 2021. (Cheng et al., 2021), Shen et al., 2022. (Shen et al., 2022), Xie et al., 2025. (Xie et al., 2025), Zhao et al., 2023. (Zhao et al., 2023), Zhang et al., 2022. (Zhang et al., 2022), Wu et al., 2010. (Wu et al., 2010), Wu et al., 2000. (Wu et al., 2000), Shen et al., 2012. (Shen et al., 2012), Liu et al., 2019. (Liu et al., 2019), and Chen and He, 2024. (Chen and He, 2024), did not provide sufficient methodological information regarding allocation concealment or the blinding of participants, investigators, and outcome assessors. Chen and He, 2024. (Chen and He, 2024) did not clearly describe how they addressed missing outcome data.

Dan L et al. (Liu et al., 2019) assigned participants using an odd-even alternation sequence rather than true randomization, resulting in a high risk of bias in randomization. Moreover, the randomization methods were insufficiently described in the studies by Cheng et al., 2021. (Cheng et al., 2021), Shen et al., 2022. (Shen et al., 2022), Wu et al., 2010. (Wu et al., 2010) and Wu et al., 2000. (Wu et al., 2000), which incompletely reported the prespecified outcomes described in their Methods sections, resulting in a judgment of “some concerns” for selective outcome reporting. All remaining domains across the included studies were assessed as having a low risk of bias.

Beyond the risk-of-bias assessment, we also evaluated the completeness of intervention reporting based on Supplementary Table S1. Batch numbers and voucher specimens were rarely reported, which may limit the traceability and full reproducibility of the herbal interventions.

Figure 2 shows the results of the risk-of-bias assessment.

**FIGURE 2:**
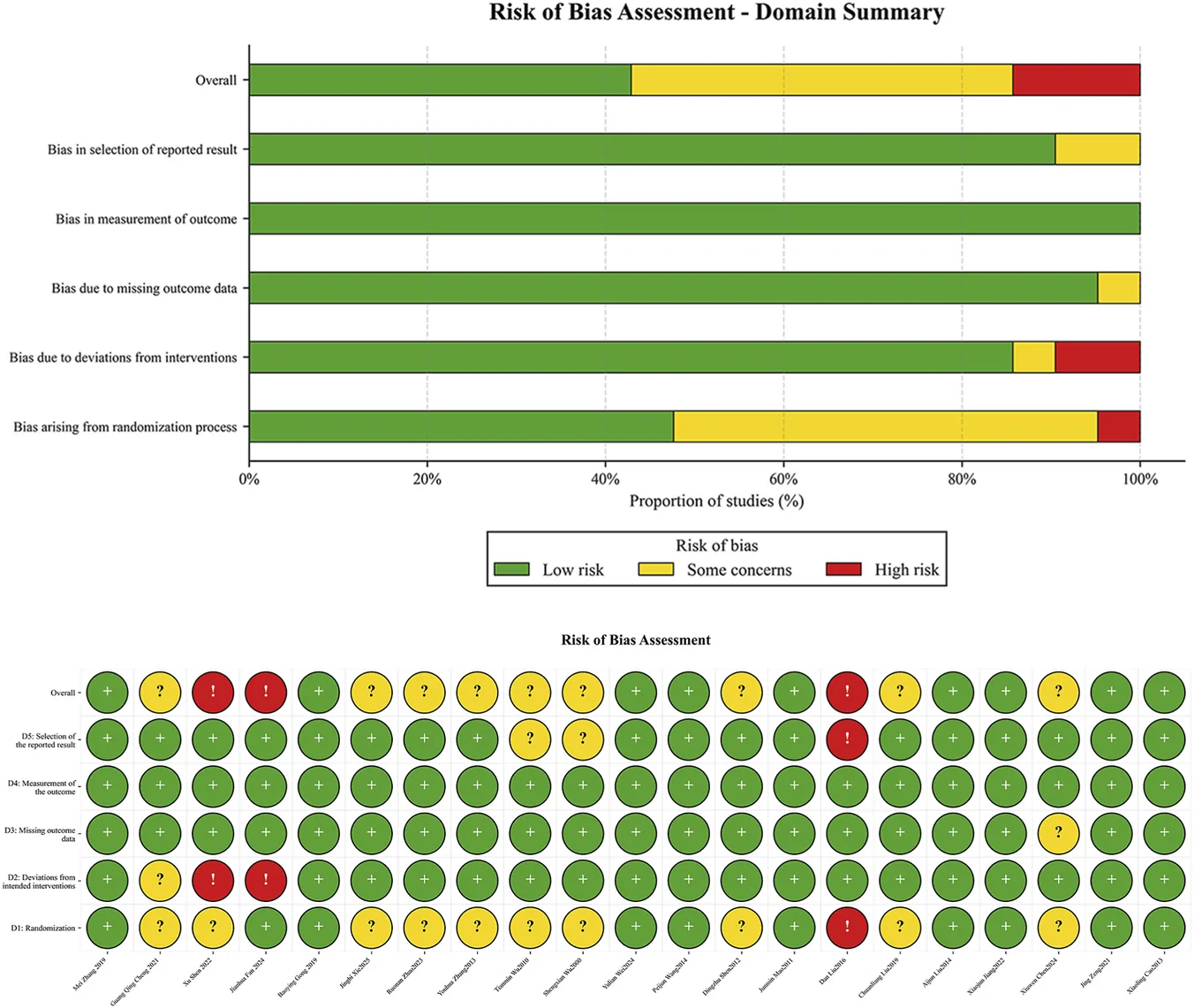
Proportion assessment diagram.

## 4 Meta-analysis

### 4.1 Evaluation of primary outcomes

#### 4.1.1 Intima-media thickness

All 21 included studies (Gong et al., 2019; Cheng et al., 2021; Fan et al., 2024; Shen et al., 2022; Zhang et al., 2019; Xie et al., 2025; Zhao et al., 2023; Zhang et al., 2022; Wu et al., 2010; Wu et al., 2000; Wei et al., 2026; Wang et al., 2014; Shen et al., 2012; Mao et al., 2011; Liu et al., 2016; Liu et al., 2019; Liu et al., 2014; Jiang and Zhang, 2022; Chen and He, 2024; Zeng et al., 2021; Cao and Wang, 2013) reported carotid IMT as an outcome measure. Considerable heterogeneity was observed across the studies (*I* ^*2*^ = 99%); therefore, a random-effects model was used for the meta-analysis. The pooled analysis demonstrated that, compared with the control group, the CHM group achieved a significantly greater reduction in IMT, with a pooled mean difference (MD) of 0.12 (95% CI: 0.07 to 0.17; *p* < 0.00001) (Figure 3).

**FIGURE 3:**
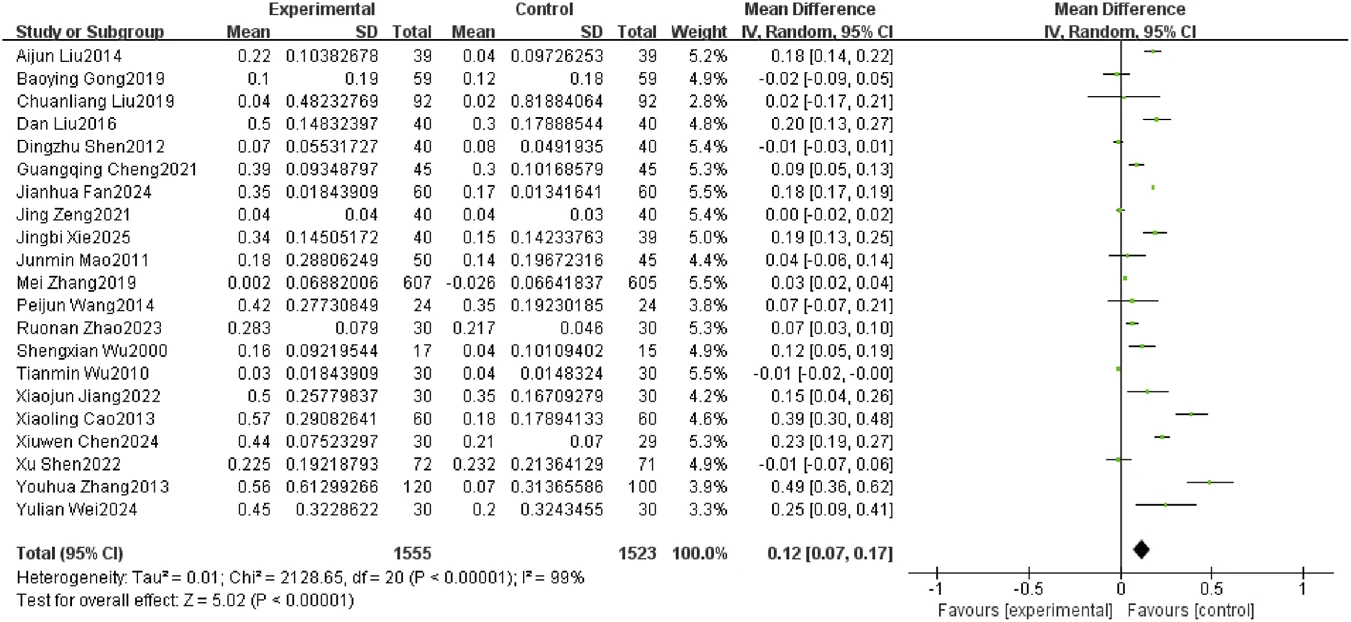
CHM showed greater IMT reduction than control.

#### 4.1.2 Comparison of plaque area

Twelve studies (31, 33, 35–40, 47–49, 51) reported carotid plaque area. Given the substantial between-study heterogeneity (I^2^ = 100%), a random-effects model was used for the meta-analysis. The pooled results showed that patients receiving CHM experienced a significantly greater reduction in carotid plaque area than those in the control group (MD = 4.94, 95% CI: 3.13 to 6.76, p < 0.01) (Figure 4).

**FIGURE 4:**
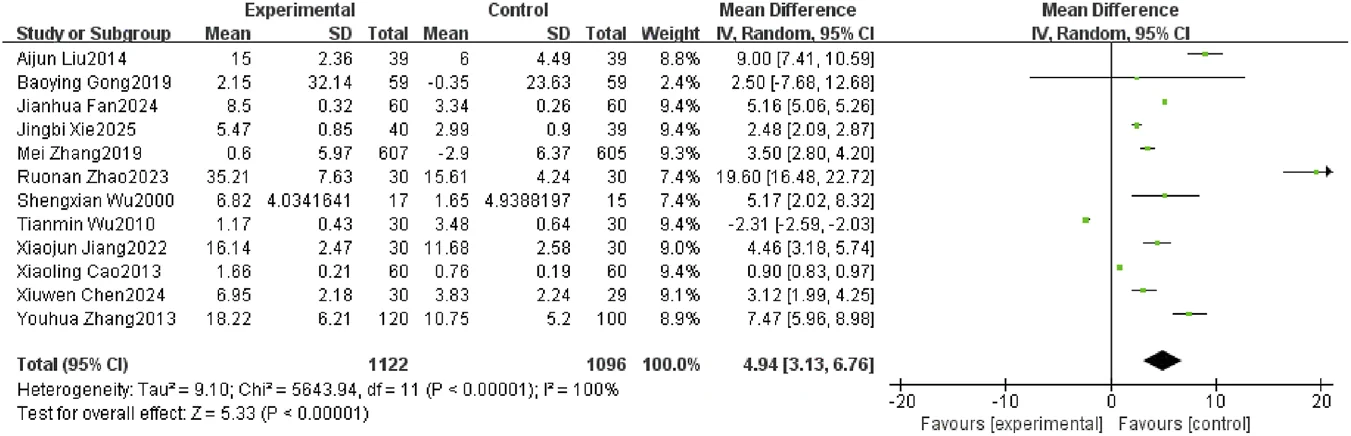
CHM showed greater plaque area reduction than control.

### 4.2 Evaluation of secondary outcomes

#### 4.2.1 Comparison of TC levels

Twenty studies (Gong et al., 2019; Cheng et al., 2021; Fan et al., 2024; Shen et al., 2022; Zhang et al., 2019; Xie et al., 2025; Zhang et al., 2022; Wu et al., 2010; Wu et al., 2000; Wei et al., 2026; Wang et al., 2014; Shen et al., 2012; Mao et al., 2011; Liu et al., 2016; Liu et al., 2019; Liu et al., 2014; Jiang and Zhang, 2022; Chen and He, 2024; Zeng et al., 2021; Cao and Wang, 2013) reported TC levels. As no significant between-study heterogeneity was detected (*I* ^*2*^ = 0%), a fixed-effects model was applied for the meta-analysis. The pooled results demonstrated that the CHM group achieved a significantly greater reduction in TC levels than the control group (MD = 0.32, 95% CI: 0.22 to 0.42, *p* < 0.00001) (Figure 5).

**FIGURE 5:**
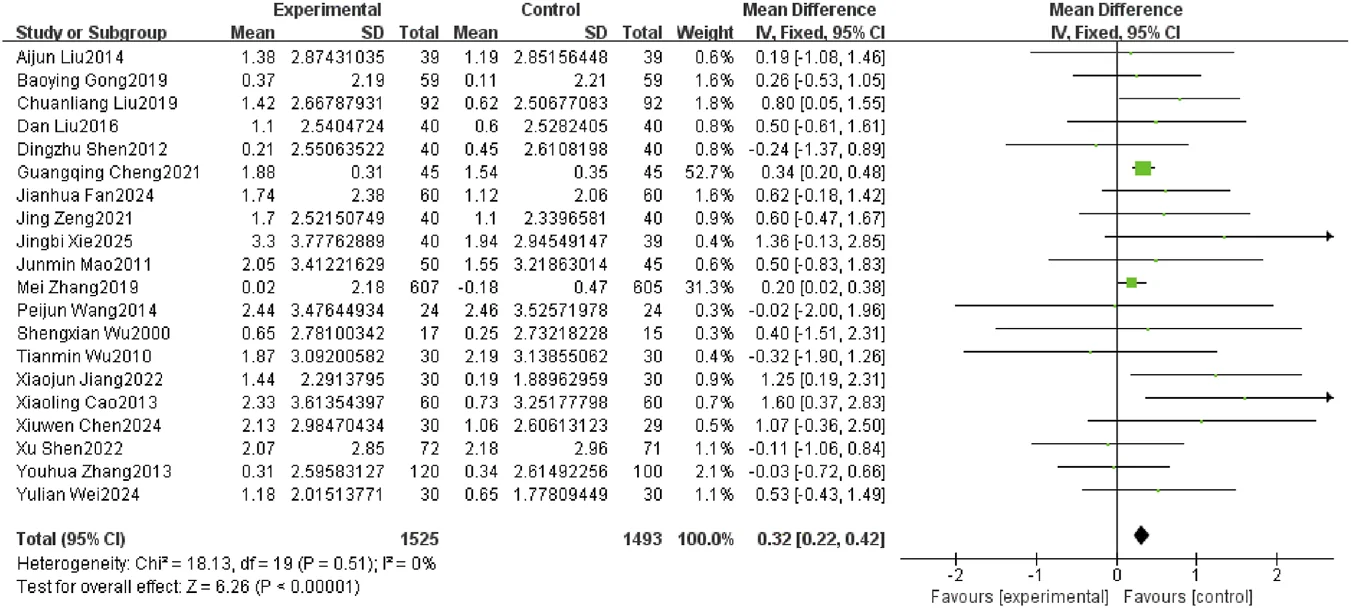
CHM showed greater TC reduction than control.

#### 4.2.2 Comparison of TG levels changes

All 21 studies (Gong et al., 2019; Cheng et al., 2021; Fan et al., 2024; Shen et al., 2022; Zhang et al., 2019; Xie et al., 2025; Zhao et al., 2023; Zhang et al., 2022; Wu et al., 2010; Wu et al., 2000; Wei et al., 2026; Wang et al., 2014; Shen et al., 2012; Mao et al., 2011; Liu et al., 2016; Liu et al., 2019; Liu et al., 2014; Jiang and Zhang, 2022; Chen and He, 2024; Zeng et al., 2021; Cao and Wang, 2013) reported TG levels. Owing to moderate between-study heterogeneity (*I* ^*2*^ = 73%), a random-effects model was used for the meta-analysis. The pooled results demonstrated that the CHM group achieved a significantly greater reduction in TG levels than the control group, with a pooled MD of 0.24 (95% CI: 0.11 to 0.37, p < 0.01) (Figure 6).

**FIGURE 6:**
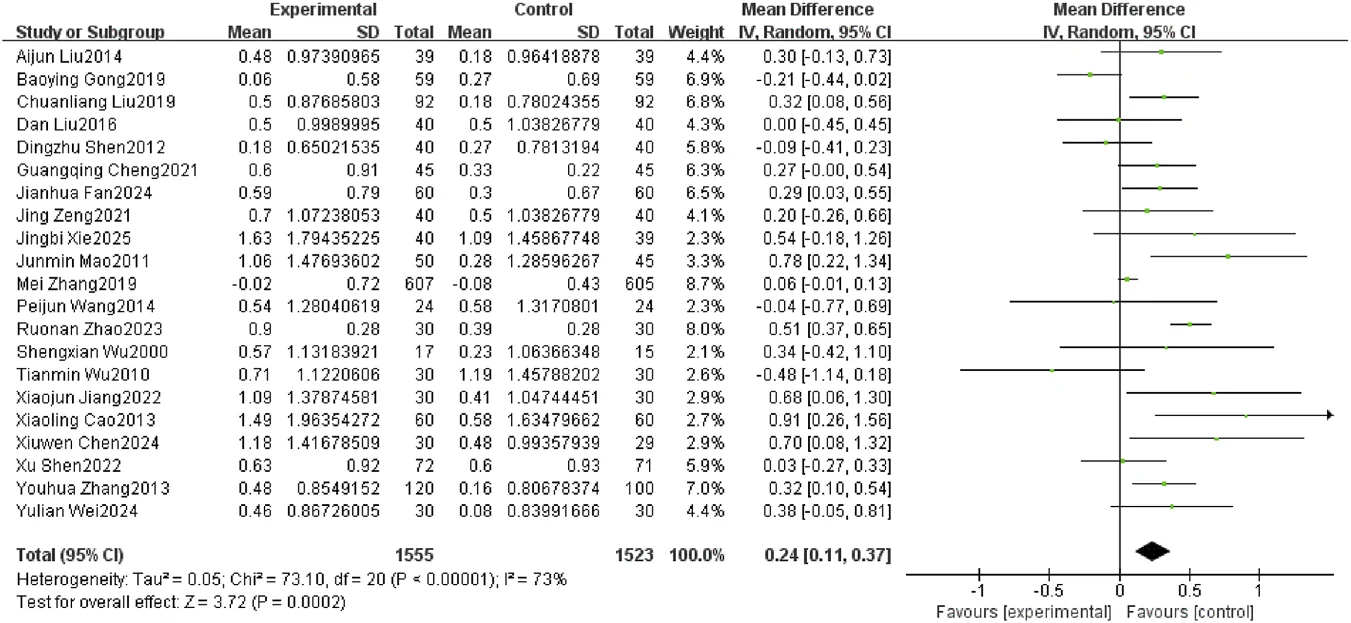
CHM showed greater TG reduction than control.

#### 4.2.3 Comparison of LDL level changes

All 21 studies (Gong et al., 2019; Cheng et al., 2021; Fan et al., 2024; Shen et al., 2022; Zhang et al., 2019; Xie et al., 2025; Zhao et al., 2023; Zhang et al., 2022; Wu et al., 2010; Wu et al., 2000; Wei et al., 2026; Wang et al., 2014; Shen et al., 2012; Mao et al., 2011; Liu et al., 2016; Liu et al., 2019; Liu et al., 2014; Jiang and Zhang, 2022; Chen and He, 2024; Zeng et al., 2021; Cao and Wang, 2013) reported changes in low-density lipoprotein cholesterol (LDL-C) levels. Considerable between-study heterogeneity was observed (*I* ^*2*^ = 97%); therefore, a random-effects model was applied for the meta-analysis. The pooled analysis showed that the CHM group achieved a significantly greater reduction in LDL-C levels than the control group (MD = 0.37, 95% CI: 0.21 to 0.53, *p* < 0.01), indicating a statistically significant treatment effect (Figure 7).

**FIGURE 7:**
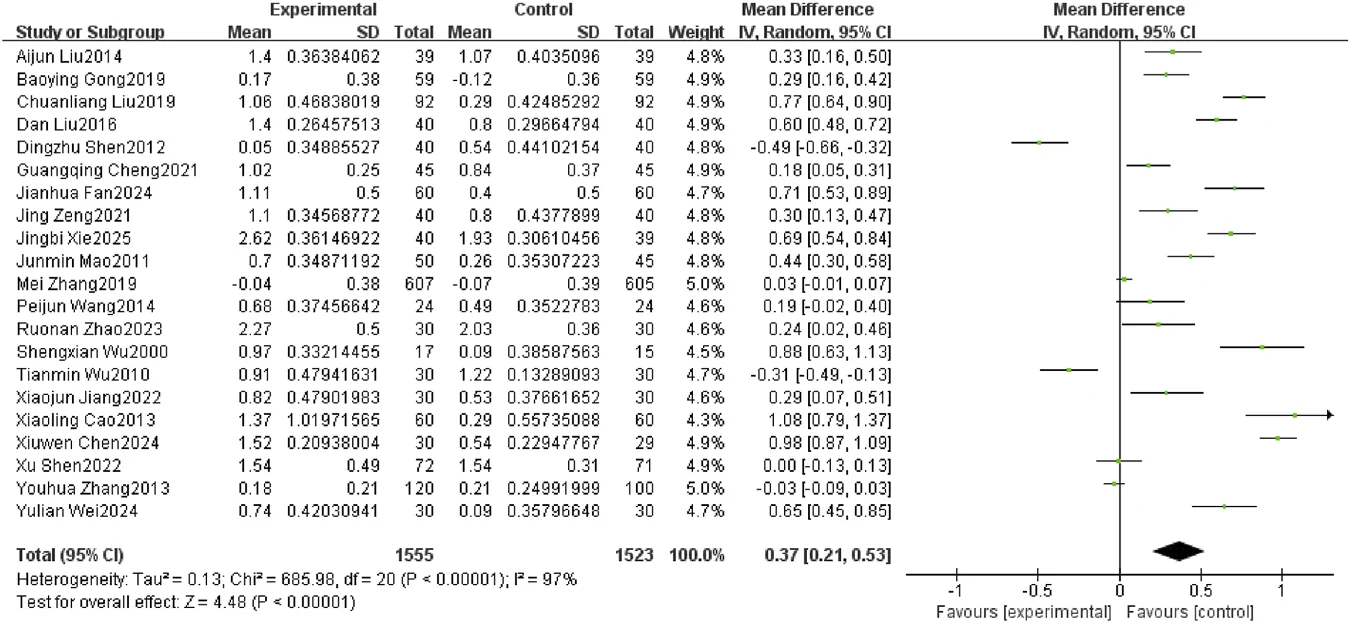
CHM showed greater LDL-C reduction than control.

#### 4.2.4 Comparison of HDL level changes

Nineteen studies (Fan et al., 2024; Shen et al., 2022; Zhang et al., 2019; Xie et al., 2025; Zhao et al., 2023; Zhang et al., 2022; Wu et al., 2010; Wu et al., 2000; Wei et al., 2026; Wang et al., 2014; Shen et al., 2012; Mao et al., 2011; Liu et al., 2016; Liu et al., 2019; Liu et al., 2014; Jiang and Zhang, 2022; Chen and He, 2024; Zeng et al., 2021; Cao and Wang, 2013) reported high-density lipoprotein cholesterol (HDL-C) levels. Owing to substantial between-study heterogeneity (*I* ^*2*^ = 98%), a random-effects model was used for the meta-analysis. The pooled results indicated that the CHM group experienced a significantly smaller reduction in HDL-C levels than the control group (MD = −0.20, 95% CI: −0.29 to −0.10, *p* < 0.01), suggesting a favorable effect of CHM on preserving HDL-C levels (Figure 8).

**FIGURE 8:**
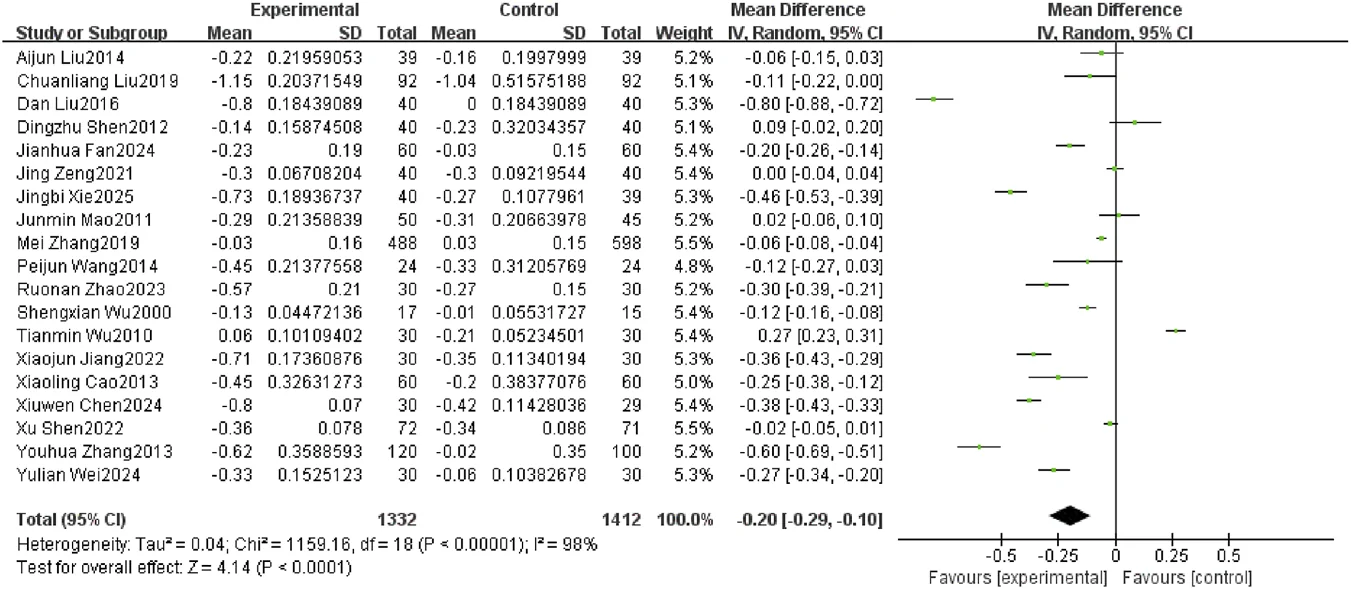
CHM showed a smaller reduction in HDL-C than control.

### 4.3 Safety indicators

Seven studies (Gong et al., 2019; Shen et al., 2022; Zhang et al., 2019; Wei et al., 2026; Mao et al., 2011; Liu et al., 2016; Cao and Wang, 2013) reported adverse events. Relative risk (RR) was used as the effect measure, and a fixed-effects model was applied because of the low level of between-study heterogeneity (*I* ^*2*^ = 29%). The pooled analysis demonstrated that CHM was not associated with a significantly increased risk of adverse events compared with the control group (RR = 0.84, 95% CI: 0.64–1.10, *p* = 0.20) (Figure 9).

**FIGURE 9:**
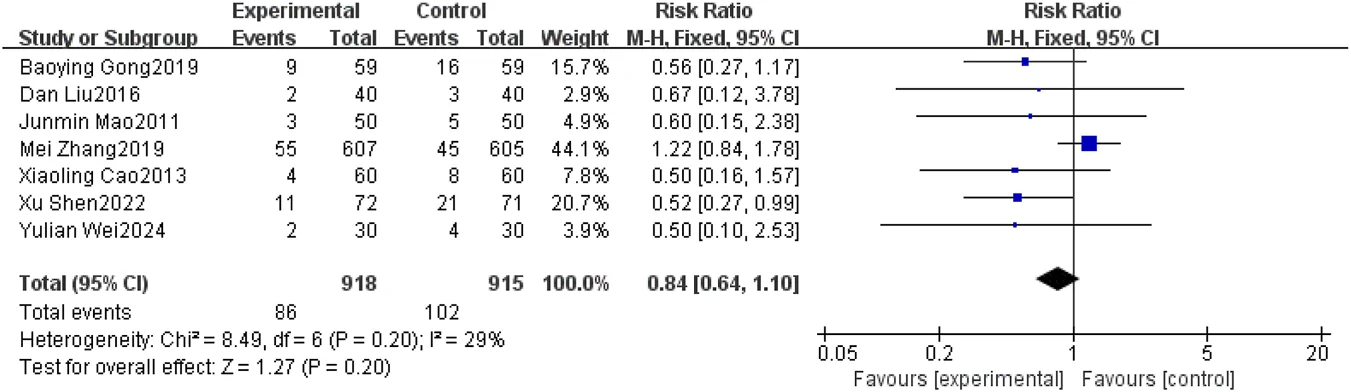
CHM did not increase AE risk versus control.

Further, 12 studies (Gong et al., 2019; Fan et al., 2024; Zhao et al., 2023; Zhang et al., 2022; Wu et al., 2010; Shen et al., 2012; Mao et al., 2011; Liu et al., 2016; Zeng et al., 2021; Cao and Wang, 2013) assessed hepatic and renal function. The findings consistently indicated that CHM treatment had no statistically significant adverse effects on liver or kidney function (*p* > 0.05), supporting its favorable safety profile.

## 5 Meta-regression analysis and exploration of sources of heterogeneity

### 5.1 Univariate meta-regression analysis

Meta-regression analysis was performed to investigate potential sources of heterogeneity using intervention type, disease severity, TCM syndrome, mean age, and treatment duration as covariates. The results identified disease severity as the principal contributor to between-study heterogeneity, accounting for 8.10% of the observed variance (R^2^ = 8.10%). The difference in treatment effect between the moderate-to-severe IMT thickening subgroup and the mild IMT thickening subgroup approached statistical significance (regression coefficient = 0.114, 95% CI: −0.019 to 0.247, *p* = 0.092). None of the remaining covariates independently explained the heterogeneity observed across the included studies.

### 5.2 Multifactorial meta-regression analysis

Variance inflation factors (VIFs) were calculated to assess multicollinearity among the covariates, including disease severity, mean age, and treatment duration. No evidence of significant multicollinearity was identified, with VIF values of 1.25 for disease severity, 1.20 for mean age, and 1.11 for treatment duration. These findings indicated that the variables could be included simultaneously in a multivariable meta-regression model. Based on the covariate with the highest R^2^ in the univariable analysis, multivariable meta-regression models were constructed by sequentially incorporating additional covariates.

Among the two-factor models, the combination of disease severity and mean age provided the greatest explanatory power (R^2^ = 14.34%), followed by disease severity combined with treatment duration (R^2^ = 13.64%) and disease severity combined with TCM syndrome pattern (R^2^ = 10.54%).

For the three-factor models, the combination of disease severity, mean age, and treatment duration demonstrated the best model performance, explaining 31.93% of the between-study heterogeneity (R^2^ = 31.93%), a substantial improvement over the best-performing two-factor model (R^2^ = 14.34%) (Figure 10). These findings suggest that baseline atherosclerotic severity, as reflected by IMT grade, together with patients’ mean age and treatment duration, jointly influences the intervention’s therapeutic efficacy, collectively accounting for nearly one-third of the heterogeneity observed across the included studies.

**FIGURE 10:**
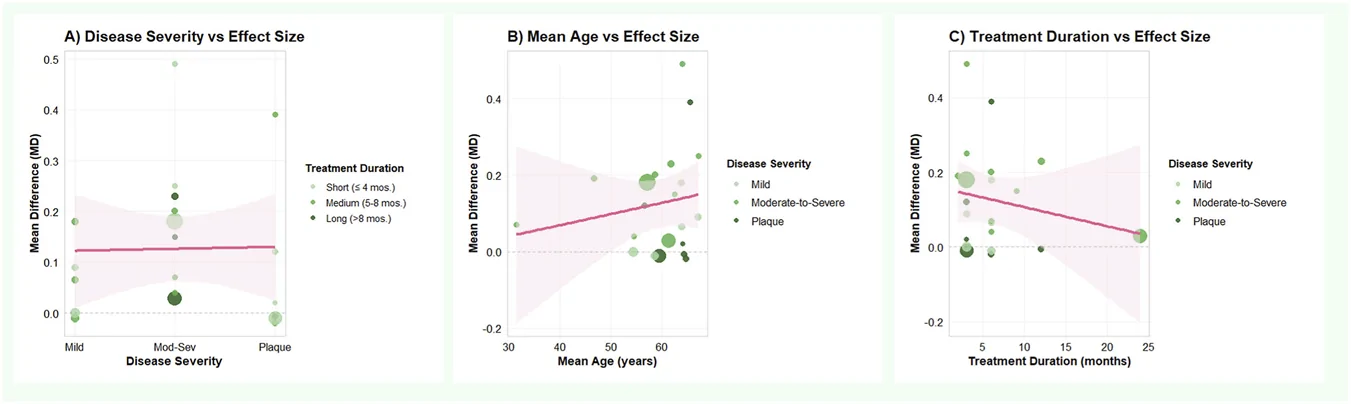
Bubble plots of univariate meta-regression analyses for three covariates: Disease severity, mean age, and treatment duration. Disease severity was the primary source of heterogeneity (R^2^ = 8.10%).

### 5.3 Subgroup analysis

Based on the meta-regression findings, subgroup analyses were performed according to disease severity, mean age, and treatment duration. To determine the optimal stratification strategy, an exhaustive search was conducted by systematically evaluating all possible age cutoffs (50–68 years) and treatment duration cutoffs (2–7 months). Despite these analyses, between-study heterogeneity remained substantial, with I^2^ values consistently exceeding 90% in most subgroup comparisons. The final subgrouping criteria were: (a) disease severity, classified as mild IMT thickening versus moderate-to-severe IMT thickening/carotid plaque; (b) mean age, ≥58 years versus <58 years; and (c) treatment duration, >3 months versus ≤3 months.

The subgroup analysis showed that Group A comprised three studies and demonstrated a pooled effect size of MD = 0.11 (95% CI: 0.05 to 0.18, *p* = 0.0009), with high between-study heterogeneity (*I* ^*2*^ = 88%). Group B, also including three studies, showed a pooled effect size of MD = 0.18 (95% CI: 0.15 to 0.21, *p* < 0.00001) and low heterogeneity (*I* ^*2*^ = 24%). Group C, consisting of eight studies, yielded a pooled effect size of MD = 0.10 (95% CI: 0.02 to 0.18, *p* = 0.01), although substantial heterogeneity persisted (*I* ^*2*^ = 95%). Group D included five studies and showed a pooled effect size of MD = 0.20 (95% CI: 0.01 to 0.39, *p* = 0.04), with similarly high heterogeneity (*I* ^*2*^ = 97%) (Figure 11).

**FIGURE 11:**
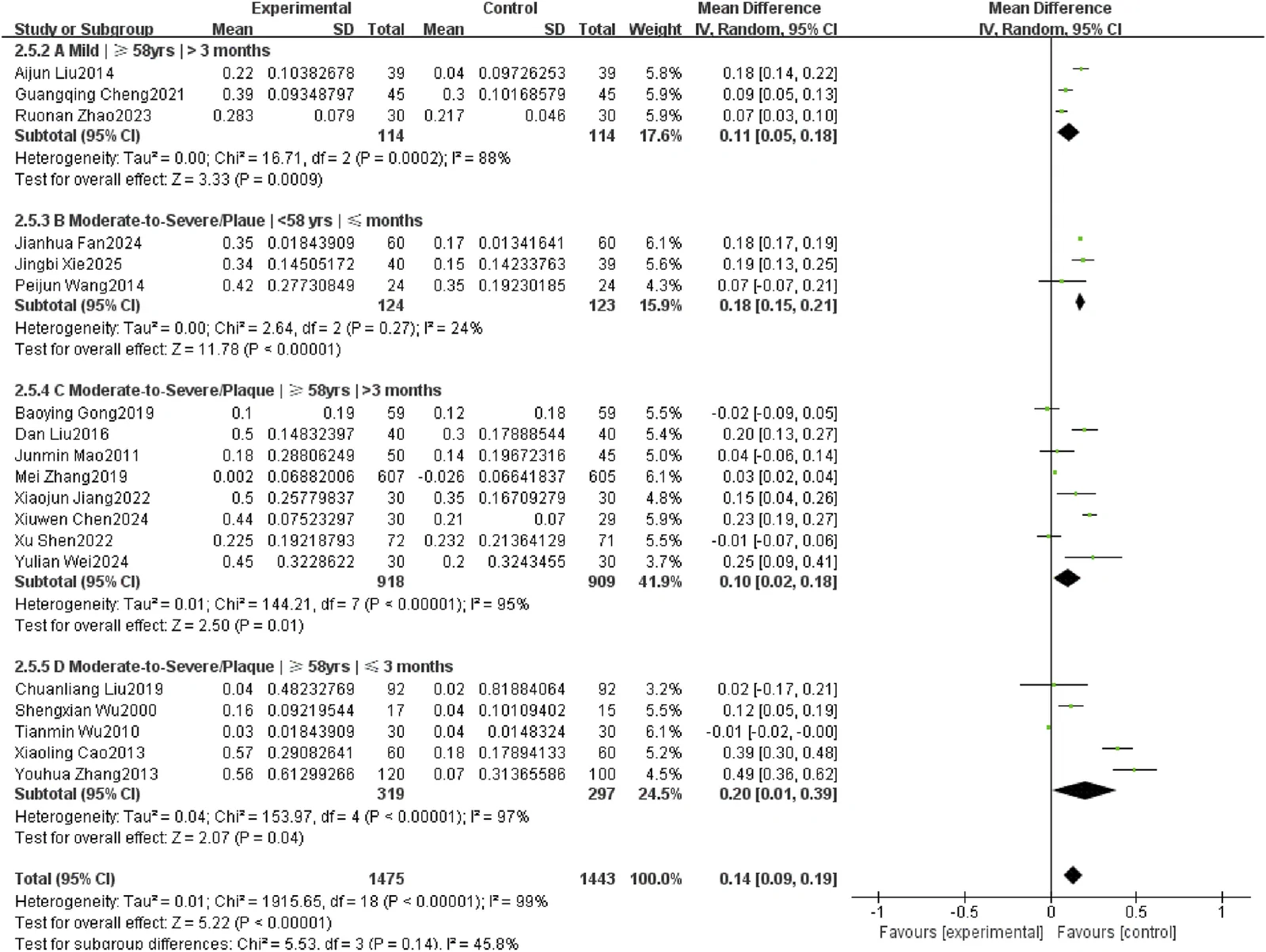
Subgroup analysis of IMT stratified by disease severity, age, and treatment duration. The pooled MDs ranged from 0.10 to 0.20 across subgroups, all favoring the CHM group.

## 6 Publication bias and sensitivity analyses

Sensitivity analyses were conducted using a leave-one-out approach to evaluate the robustness of the pooled results. Sequentially excluding each study did not materially alter the overall effect estimates, indicating that the findings were stable and not disproportionately influenced by any single study. A funnel plot was constructed to assess potential publication bias for changes in TC before and after treatment. The plot appeared largely symmetrical, suggesting no evident publication bias among the included studies (Figure 12).

**FIGURE 12:**
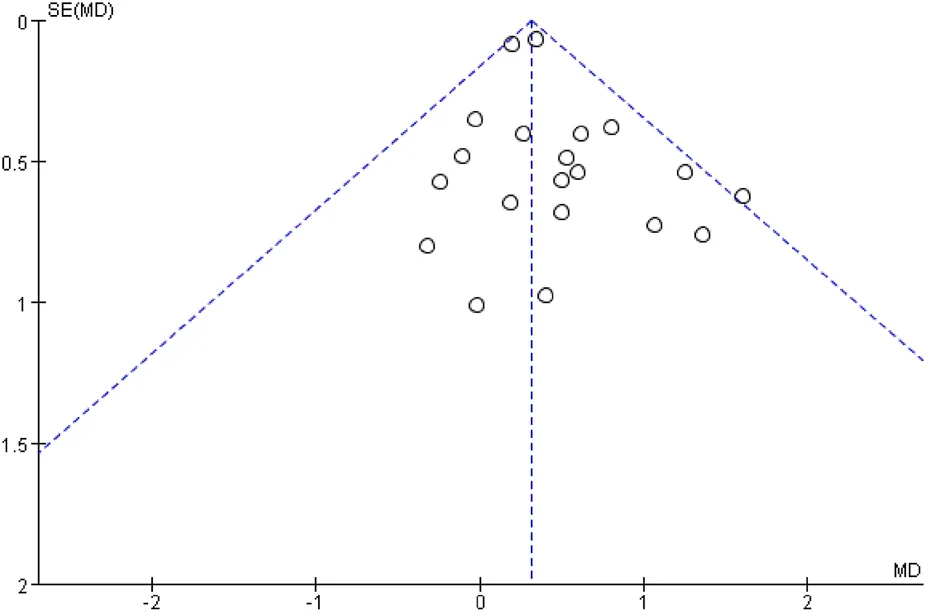
The symmetrical distribution suggests no significant publication bias.

## 7 Exploration of medication patterns

All 21 studies used compound Chinese botanical formulations, consisting of 93 distinct botanical drugs with a total usage frequency of 171 instances. Among these, 35 medicines were repeatedly prescribed, accounting for 75 repeated occurrences (Supplementary Table S3). The most frequently used botanical medicines were *Danshen* (*Salvia miltiorrhiza* Bunge.) and *Banxia* [*Pinellia ternata* (Thunb.)Makino].

To explore the underlying medication patterns across the included studies, the pharmacological actions of the prescribed formulas and their constituent botanical drugs were extracted, followed by network topology analysis (Figure 13). In the formula–pharmacological action network, clear phlegm (Degree = 10), removing blood stasis (Degree = 7), and activating blood circulation (Degree = 7) emerged as core nodes, representing the principal therapeutic pathways. In the ormula–botanical drug–pharmacological action network, *Danshen* (Degree = 34) and *Banxia* (Degree = 24) occupied key hub positions. Their primary attributed pharmacological actions were associated with removing blood stasis (Degree = 33) and regulating lipids (Degree = 28).

**FIGURE 13:**
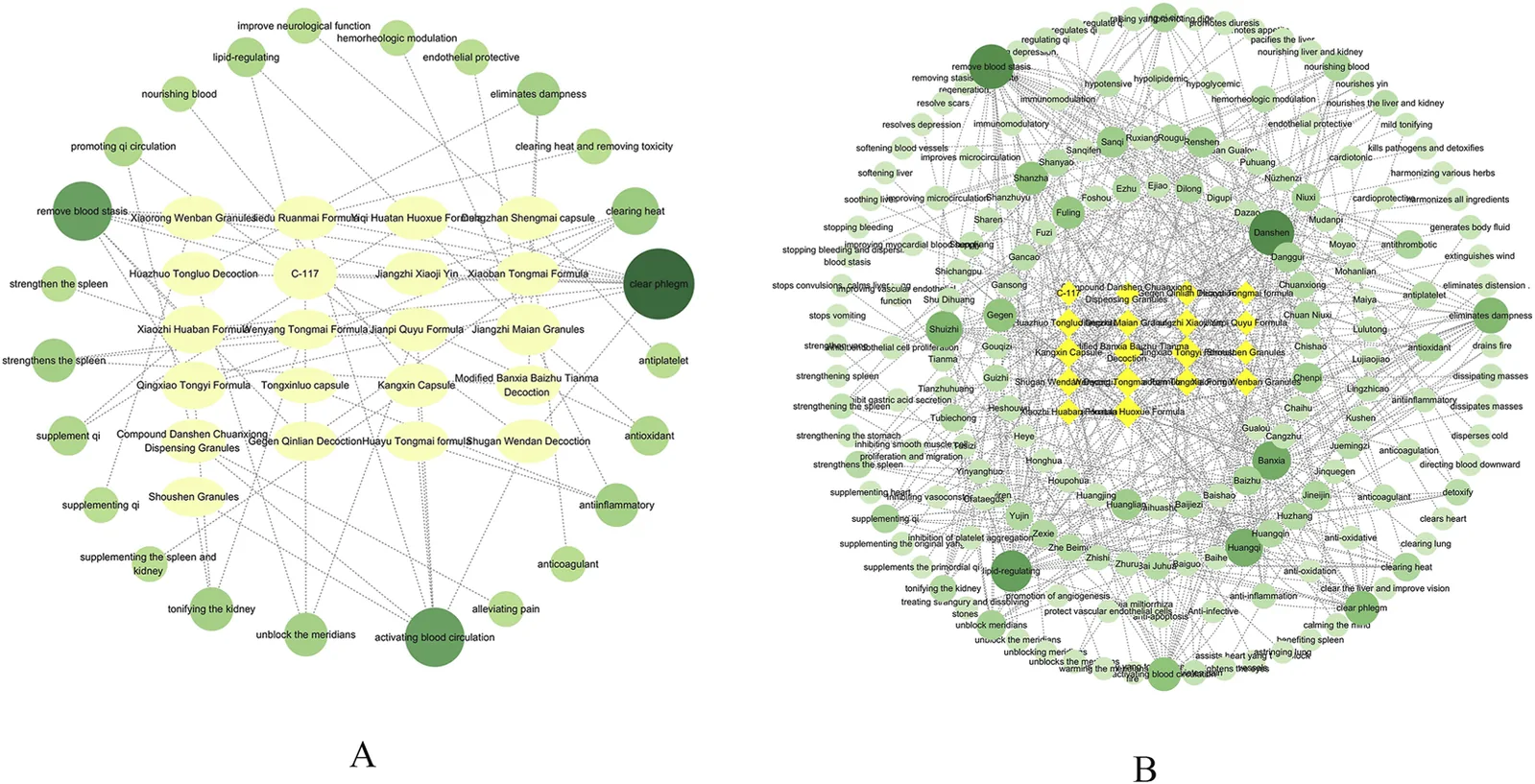
Formula-pharmacological action **(A)** and formula-botanical drug-efficacy **(B)** association networks. Yellow nodes represent TCM formulas; green nodes represent constituent botanical drugs and their pharmacological efficacies. Node size indicates degree centrality (larger nodes are more central). Edges indicate botanical drug-action correlations, with line density reflecting overall regulatory complexity. Detailed botanical drug information is listed in Supplementary Table S1.

## 8 Discussion

### 8.1 Efficacy and safety

Previous studies have shown that selected CHM preparations may improve CAS by reducing serum lipid levels and attenuating carotid plaque progression (Song and Chen, 2021; Huang et al.). Building on this evidence, a systematic review and meta-analysis of RCTs were performed to comprehensively evaluate the efficacy and safety of selected CHM preparations for CAS. The findings indicate that, when used as an adjunct to conventional therapy, selected CHM preparations may further reduce carotid IMT and plaque area while improving lipid metabolism. Substantial evidence links both IMT and carotid plaque burden to cardiovascular risk, supporting the clinical significance of these effects. Mitra S et al. (Mitra et al., 2025) demonstrated a linear increase in cardiovascular event risk with increasing IMT.

In comparison, Spence JD et al. (Spence et al., 2002) reported that larger carotid plaque areas are associated with a greater likelihood of future cardiovascular events. Therefore, the additional reductions in IMT and plaque burden observed with adjunctive selected CHM preparations may translate into clinically meaningful cardiovascular benefits beyond those achieved with conventional therapy alone. These results are consistent with previous systematic reviews and clinical studies (Hu et al., 2022b; Roghani-Shahraki et al., 2021).

The negative mean difference observed for HDL-C (MD = −0.20) should not be interpreted as a detrimental effect of CHM. Because treatment effects were calculated as the difference between pre-intervention and post-intervention values, a negative MD indicates that the CHM group experienced a smaller decline in HDL-C than the control group, reflecting a protective effect on HDL-C levels. This observation is biologically plausible and is consistent with previous studies showing that ginsenosides and salvianolic acids can enhance HDL metabolism by promoting ATP-binding cassette transporter A1 (ABCA1)-mediated reverse cholesterol transport (Sun et al., 2024).

Our safety analysis further demonstrated that the selected CHM regimens did not significantly affect hepatic or renal function and were not associated with an increased incidence of adverse events. These findings agree with previous reports (Duan et al., 2023; Li et al., 2022b). This favorable safety profile is particularly relevant in clinical practice, given the well-recognized risk of hepatotoxicity associated with intensive statin therapy. However, caution remains warranted because certain botanical drug ingredients in some formulations have inherent toxic properties (Chinese Pharmacopoeia Commission, 2025), and berberine has also shown potential toxic effects in recent experimental studies (Duan et al., 2023). Moreover, the complex multi-component nature of CHM formulations presents considerable challenges for toxicological evaluation and limits comprehensive assessment of their real-world safety. Therefore, further high-quality research focusing on the safety profiles, toxicity mechanisms, and long-term adverse effects of Chinese herbal medicines is essential to support their broader clinical application and evidence-based use (Liu et al., 2022; Yao, 2026; Zhong et al., 2023).

### 8.2 Exploration of medication patterns

The analysis showed that compound formulas with blood stasis-removing and phlegm-clearing effects were the most commonly used treatments for CAS, consistent with previous studies (Liang et al., 2025; Zhang et al., 2023; Qiang et al., 2026). *Danshen* and *Banxia* emerged as core botanical drugs, characterized by high usage frequencies and central positions within the medication network, further supporting previous findings (Wang and Zhang, 2024; Lu et al., 2023; Lu et al., 2020; Zou et al., 2023; Lee et al., 2021). *Danshen* extracts have been reported to downregulate the expression of vascular cell adhesion molecule-1 (VCAM-1) and intercellular adhesion molecule-1 (ICAM-1), while reducing the production of pro-inflammatory cytokines, including tumor necrosis factor-α (TNF-α) and interleukin-6 (IL-6). These effects may improve vascular function and attenuate the progression of carotid atherosclerosis (Wang and Zhang, 2024; Lu et al., 2023). Banxia may exert lipid-regulating effects by upregulating adipose triglyceride lipase (ATGL) and hormone-sensitive lipase (HSL) expression to promote lipolysis, while activating LC3, ATG5, and ATG7 to initiate lipophagy and accelerate lipid droplet degradation (Lu et al., 2020; Zou et al., 2023; Lee et al., 2021).

### 8.3 Limitations and sources of heterogeneity

#### 8.3.1 Sources of heterogeneity

Most pooled effect estimates in this meta-analysis showed substantial between-study heterogeneity. Although meta-regression and subgroup analyses were conducted to identify potential sources of heterogeneity, the final multivariable model explained only 31.93% of the observed variability (R^2^ = 31.93%), indicating that a considerable proportion of the heterogeneity remains unexplained. Careful examination of the included studies revealed marked differences in botanical drug formulation composition, compatibility principles, dosages, administration methods, and decoction procedures, all of which may have contributed to the residual heterogeneity.

Future clinical trials should adopt standardized treatment protocols and report botanical drug formulations, preparation methods, dosing regimens, and other key methodological parameters in detail to improve comparability. Moreover, when feasible, individual participant data meta-analysis (IPD-MA) should be considered, as it enables more accurate adjustment for patient-level confounding factors and a more comprehensive exploration of heterogeneity, generating stronger and more reliable evidence to inform clinical practice.

#### 8.3.2 Generalizability and safety of the findings

All studies included in this meta-analysis enrolled patients with confirmed carotid atherosclerosis, and the CHM preparations evaluated represent only a limited subset of the extensive range used in clinical practice. Therefore, these findings should not be interpreted as evidence supporting the efficacy of all CHM preparations for carotid atherosclerosis. Instead, they suggest that botanical drug formulations sharing the common medication patterns identified in this analysis may offer therapeutic benefits. Caution is warranted when extrapolating these results to other botanical drug formulations that were not evaluated in the included studies or to broader populations, including individuals with asymptomatic carotid atherosclerosis. Moreover, given the inherent bioavailability limitations of oral CHM preparations, interpret our exploratory findings with caution and require further mechanistic and pharmacokinetic validation.

#### 8.3.3 Overlapping botanical drugs’ impact

All included studies evaluated compound Chinese botanical formulations rather than individual botanical drugs, and the overlap of constituent botanical drugs across formulations should be considered when interpreting the pooled findings. Our aggregate analysis of the prescriptions used in the included studies identified 93 distinct botanical medicines, of which 35 were repeatedly prescribed, accounting for 75 repeated occurrences (Supplementary Table S3). The frequent recurrence of these shared botanical medicines (or their bioactive metabolites) may contribute to the consistent therapeutic effects of selected CHM preparations observed in patients with CAS.

However, because CHM formulations exert therapeutic effects through complex multi-component, multi-target, and multi-pathway interactions, different botanical drug combinations may produce distinct pharmacological effects and influence different biological targets. Based on the currently available evidence, it is not possible to determine whether the observed clinical benefits arise primarily from individual botanical drugs, common bioactive metabolites, or synergistic interactions among multiple botanical drug components. Similarly, the available data do not permit attribution of the favorable safety profile to any specific botanical drug or constituent. Future studies integrating comprehensive phytochemical characterization, mechanistic pharmacological investigations, network pharmacology, and rigorously designed, standardized clinical trials are needed to clarify the contributions of overlapping botanical components and their synergistic interactions in treating carotid atherosclerosis.

#### 8.3.4 Lack of long-term safety data

Most of the included RCTs did not systematically evaluate or report long-term safety outcomes, reflecting a common limitation in research on CHM preparations. Future clinical trials should follow the CONSORT Extension for Chinese Herbal Medicine Formulas to improve the completeness, consistency, and transparency of safety reporting, including comprehensive documentation of adverse events and long-term safety outcomes.

## 9 Conclusion

This study suggests that selected CHM preparations may effectively reduce carotid IMT, decrease carotid plaque area, and improve lipid metabolism in patients with CAS, without increasing the risk of adverse events or adversely affecting hepatic or renal function. However, well-designed studies with rigorous methodologies and standardized safety assessments should further confirm the safety of these interventions. Network topology analysis further revealed that the core therapeutic approach operates through phlegm-resolving, blood-stasis-removing, and blood-activating actions, with Danshen and Banxia serving as the pivotal botanical drug hubs.

## Data Availability

The original contributions presented in the study are included in the article/Supplementary Material, further inquiries can be directed to the corresponding authors.

## Glossary

CAS: Carotid atherosclerosis
IMT: Increased intima–media thickness
LDL: Low-density lipoprotein triglycerides
TC: Total cholesterol
HDL-C: High-Density Lipoprotein Cholesterol
TCM: Traditional Chinese medicine
CHM: Chinese herbal medicine
RCTs: Randomized controlled trials
AE: Adverse events
MeSH: Medical Subject Headings
RR: Relative risk
MD: Mean difference
SMD: Standardized mean difference
CIs: Confidence intervals
TXL: Tongxinluo capsule
HYTM: Huayu Tongmai formula
DZSM: Dengzhan Shengmai capsule
SS: Shoushen granule
JZMA: Jiangzhi Maian Granules
ASA: Aspirin Enteric-Coated Tablets
ATV: Atorvastatin Calcium Tablets
ISMN: Isosorbide Mononitrate Sustained-Release Tablets
PRV: Pravastatin Sodium
CPA: Carotid artery plaque area
AE: Adverse Events
VCAM-1: Vascular Cell Adhesion Molecule-1
ICAM-1: Intercellular Cell Adhesion Molecule-1
TNF-α: Tumor Necrosis Factor-alpha
IL-6: Interleukin-6
SIRT1/PPARγ: Peroxisome Proliferator-Activated Receptor Gamma

### Validated list of medicinal plant species cited in this study

Scorpion: *Buthus martensii* Karsch
Centipede: *Scolopendra subspinipes mutilans,* L. Koch
Panax: *Panax ginseng* C. A. Mey
Licorice: *Glycyrrhiza uralensis* Fisch
Salvia: *Salvia miltiorrhiza* Bge
Astragalus membranaceus: *Astragalus membranaceus*(Fisch.)Bge

## References

[B1] Adhyaru B. B. Jacobson T. A. (2018). Safety and efficacy of statin therapy. Nat. Rev. Cardiol. 15, 757–769. 10.1038/s41569-018-0098-5 30375494

[B2] Almevall A. D. Wennberg P. Liv P. Nyman E. Lindvall K. Norberg M. (2025). Midlife mediterranean diet is associated with subclinical carotid atherosclerosis in late midlife. Eur. J. Prev. Cardiol. 32, 1614–1628. 10.1093/eurjpc/zwaf155 40100758

[B3] Cao X. L. Wang Y. , (2013). Clinical observation of Qi-replenishing,blood-activating and phlegm-removing therapy in the treatment of carotid atherosclerotic plaques. J. Sichuan Traditional Chin. Med, 31(12):69–72. 10.3969/j.issn.1000-3649.2013.12.025

[B4] Cerebrovascular Disease Society, Chinese Society of Neurology (2017). Consensus on diagnosis and treatment of cerebral and carotid atherosclerosis in China. Chin. Soc. Neur 50, 572–578. 10.3760/cma.j.issn.1006-7876.2017.08.003

[B5] Chang Y. W. Fan L. Sun G. T. Yang C. Y. Liang X. W. Zhang Y. (2025). A retrospective cohort study on the clinical efficacy of shanhuang jiangzhi tablets and atorvastatin calcium tablets in the treatment of carotid atherosclerosis. J. Health Popul. Nutr. 44, 324. 10.1186/s41043-025-01057-0 PMC12465801 40999530

[B6] Chen X. W. He L. R. , (2024). Clinical observation on qingxiao tongyi formula in the treatment of carotid artery plaque with phlegm-stasis intermingling syndrome[J]. Guangming J. Chin. Med, 39(13): 2633–2636. 10.3969/j.issn.1003-8914.2024.13.028

[B7] Cheng G. Yan X. Wang F. Chen C. (2021). Correlation analysis of huayu tongmai decoction intervention and prognosis indexes of patients with carotid atherosclerosis. Evid. Based Complement. Altern. Med. 2021, 2739092–2739097. 10.1155/2021/2739092 PMC8898143 35256887

[B8] Chinese Pharmacopoeia Commission (2025). Pharmacopoeia of the People's Republic of China: Volume I. 2025. Edition. Beijing: China Medical Science and Technology Press.

[B9] Chinese Society of Health Management, Chinese Society of Ultrasound in Medicine, Chinese Society of Cardiology, and Editorial Board of Chinese Journal of Health Management (2015). Guidelines for carotid ultrasound examination in the Chinese health–examination population. Chin. J. Health Manag. 9, 254–260. (in Chinese). 10.3760/cma.j.issn.1674-0815.2015.04.004

[B10] Duan H. Song P. Li R. Su H. He L. (2023). Attenuating lipid metabolism in atherosclerosis: the potential role of anti-oxidative effects on low-density lipoprotein of herbal medicines. Front. Pharmacol. 14, 1161657. 10.3389/fphar.2023.1161657 PMC10102431 37063287

[B11] Fan J. Yu C. Tang H. Zhang Z. Wu Q. Lu L. (2024). Efficacy of Jiangzhi Mai’an granules in the treatment of stable angina pectoris with phlegm and blood stasis obstruction and carotid artery plaque: a prospective randomized study. Medicine 103, e40787. 10.1097/MD.0000000000040787 PMC11630999 39654241

[B12] Fu J. Deng Y. Ma Y. Man S. Yang X. Yu C. (2024). National and provincial-level prevalence and risk factors of carotid atherosclerosis in Chinese adults. JAMA Netw. Open 7, e2351225. 10.1001/jamanetworkopen.2023.51225 PMC10784858 38206625

[B13] Gong B. Chen X. Lin R. Zhang F. Zhong J. Zhang Q. (2019). Safety and efficacy of the C‐117 formula for vulnerable carotid artery plaques (spchim): a randomized double‐blind controlled pilot study. Evid. Based Complement. Altern. Med. 2019, 9746492. 10.1155/2019/9746492 31391862 PMC6662507

[B14] Gresele P. Paciullo F. Migliacci R. (2020). Antithrombotic treatment of asymptomatic carotid atherosclerosis: a medical dilemma. Intern Emerg. Med. 15, 1169–1181. 10.1007/s11739-020-02347-7 32405817

[B15] Guo C. Li M. Wang L. Bai Y. Rong Y. Zhang M. (2025). A retrospective cross-sectional study of association between triglyceride-glucose index and carotid atherosclerosis. Front. Cardiovasc Med. 12, 1611466. 10.3389/fcvm.2025.1611466 PMC12179170 40547502

[B16] Hald E. M. Lijfering W. M. Mathiesen E. B. Johnsen S. H. Løchen M. L. Njølstad I. (2014). Carotid atherosclerosis predicts future myocardial infarction but not venous thromboembolism: the Tromsø study. Arterioscler. Thromb. Vasc. Biol. 34, 226230. 10.1161/ATVBAHA.113.302162 24177328

[B17] Hu X. Hu Y. Sun X. Li Y. Zhu Y. (2022a). Effect of aspirin in patients with established asymptomatic carotid atherosclerosis: a systematic review and meta-analysis. Front. Pharmacol. 13, 1041400. 10.3389/fphar.2022.1041400 PMC9768439 36569309

[B18] Hu X. Liu J. Li W. Wang C. Li G. Zhou Y. (2022b). Elevated serum uric acid was associated with pre-inflammatory state and impacted the role of HDL-C on carotid atherosclerosis. Nutr. Metab. Cardiovasc Dis. 32, 1661–1669. 10.1016/j.numecd.2022.03.026 35469728

[B19] Huang Y. W. Yang Y. Y. Wu X. D. Song X. Yang Y. T. Luo S. F. , Study on the effect of salvianolic acid B in intervening atherosclerosis through the mitochondrial apoptosis pathway. Mod. Med. J. China 26, 22–27. (in Chinese). 10.3969/j.issn.1672-9463.2024.06.004

[B20] Huang J. Liu C. Jiao S. Chen Y. Xu L. Gong T. (2025). Application of high-resolution MRI in evaluating Statin efficacy on symptomatic intracranial atherosclerosis. Eur. Radiol. 35, 441–452. 10.1007/s00330-024-10968-1 39030372

[B21] Jia J. Zhang L. Wang L. Ji C. Xia R. Yang Y. (2021). A systematic review and meta-analysis on the efficacy of statins in the treatment of atherosclerosis. Ann. Palliat. Med. 10, 6793–6803. 10.21037/apm-21-1243 34237978

[B22] Jiang X. J. Zhang B. , (2022). Efficacy observation of wenyang tongmai formula on carotid atherosclerotic plaque with syndrome of cold-coagulation in blood vessel[J]. Shanxi J. Tradit. Chin. Med, 38(7): 12–14. 10.20002/j.issn.1000-7156.2022.07.005

[B23] Jiang L. Fan W. Zhou F. Liu L. Pan M. Yang Q. (2025). Comparative efficacy of eight traditional Chinese medicines combined with statins in the treatment of hyperlipidemia: a Bayesian network meta-analysis. Front. Pharmacol. 16, 1614767. 10.3389/fphar.2025.1614767 PMC12361126 40837400

[B24] Kadoglou N. P. Khattab E. Velidakis N. Patsourakos N. Lambadiari V. (2022). A new approach of statin therapy in carotid atherosclerosis: targeting indices of plaque vulnerability on top of lipid-lowering. A narrative review. Kardiol. Pol. 80, 880–890. 10.33963/KP.a2022.0155 35734817

[B25] Lee H. Kim M. H. Jin S. C. Han J. M. Park J. H. Yang W. M. (2021). Lipolytic and lipophagic effects of Pinellia ternata pharmacopuncture on localized adiposity. Evidence-Based Complementary Altern. Med. 21, 7347639. 10.1155/2021/7347639 PMC7806390 33505503

[B26] Li S. Gao Y. Ma K. Li Y. Liu C. Yan Y. (2021). Lipid-related protein NECTIN2 is an important marker in the progression of carotid atherosclerosis: an intersection of clinical and basic studies. J. Transl. Int. Med. 9, 294–306. 10.2478/jtim-2021-0044 PMC8802405 35136728

[B27] Li W. Wang Y. Chen S. Zhao J. Su Q. Fan Y. (2022a). Evaluation of carotid artery atherosclerosis and arterial stiffness in cardiovascular disease risk: an ongoing prospective study from the kailuan cohort. Front. Cardiovasc Med. 9, 812652. 10.3389/fcvm.2022.812652 PMC9108697 35586658

[B28] Li J. Du Y. Cai C. Liu F. (2022b). Effectiveness and safety of treating carotid atherosclerotic plaques with the method of nourishing qi, promoting blood circulation and expelling phlegm: a systematic review and meta-analysis. Front. Pharmacol. 13, 1059737. 10.3389/fphar.2022.1059737 PMC9682067 36438842

[B29] Liang Y. Q. Liu J. Zhang C. Cheng J. Lu F. Chen P. (2025). Integrating serum pharmacochemistry and metabolomics to reveal the potential effective ingredients and mechanism of huangqi chifeng tang intervening carotid atherosclerosis. J. Ethnopharmacol. 351, 120065. 10.1016/j.jep.2025.120065 40456475

[B30] Liu A. J. Dong H. W. An L. Z. Jiang H. Kan X. L. Zhao S. Q. (2014). Effect of huazhuo Tongluo decoction on intima-media thickness and atherosclerotic plaques in patients with carotid atherosclerosis. Sichuan J. Traditional Chin. Med. 32 (11), 67–68.

[B31] Liu D. Xu X. J. Tang X. Z. (2016). Clinical observation of jianpi quyu decoction in treating carotid atherosclerosis. Sichuan J. Tradit. Chin. Med. 34 (2), 81–83.

[B32] Liu C. L. Chen G. H. Li L. , (2019). Study on the effect of compound danshen chuanxiong herbal formula granules on carotid atherosclerosis in elderly patients. World Latest Med. Inf. Abstr. 19(82):15–16. 10.19613/j.cnki.1671-3141.2019.82.005

[B33] Liu H. Zhu L. Chen L. Li L. (2022). Therapeutic potential of traditional Chinese medicine in atherosclerosis: a review. Phytother. Res. 36, 4080–4100. 10.1002/ptr.7590 36029188

[B34] Lu H. K. Huang Y. Liang X. Y. Dai Y. Y. Liu X. T. (2020). Pinellia ternata attenuates carotid artery intimal hyperplasia and increases endothelial progenitor cell activity via the PI3K/Akt signalling pathway in wire-injured rats. Pharm. Biol. 58 (1), 1193–1200. 10.1080/13880209.2020.1845748 33253601 PMC7717851

[B35] Lu Y. X. Sun Y. M. Sun Y. (2023). Effects of danggui shaoyao San Decoction and its different ingredient combinations on platelet aggregation rate and endothelial cell adhesion molecules in ApoE^-^/^-^ mice with atherosclerosis. Glob. Tradit. Chin. Med. 16, 1067–1073. (in Chinese). 10.3969/j.issn.1674-1749.2023.06.002

[B36] Ma Z. Zhang M. (2025). Research progress on pharmacological effects of traditional Chinese medicine pairs and their active components against atherosclerosis. Drug Eval. Res. 48, 3737–3755. (in Chinese). 10.7501/j.issn.1674-6376.2025.12.029

[B37] Mao J. M. Ying W. J. Shi Z. D. Lin L. ,(2011). Study on clinical intervention of jiangzhixiaojiedecotion on carotid artery atherosclerosis[J]. Mod. J. Integr. Traditional Chin. West. Med. 20(13):1574–1576. 10.3969/j.issn.1008-8849.2011.13.002

[B38] Mitra S. Biswas R. K. Hooijenga P. Cassidy S. Nova A. De Ciutis I. (2025). Carotid intima-media thickness and cardiometabolic risk biomarkers in 29,000 UK biobank adults: associations with major adverse cardiovascular events. Am. J. Prev. Cardiol. 22, 101011. 10.1016/j.ajpc.2025.101011 40510259 PMC12162042

[B39] Motomiya M. Karino T. (1984). Flow patterns in the human carotid artery bifurcation. Stroke 15, 50–56. 10.1161/01.str.15.1.50 6695430

[B40] Nai W. Lei L. Zhang Q. Yan S. Xu J. Lin L. (2025). Systemic inflammation response index and carotid atherosclerosis incidence in the Chinese population: a retrospective cohort study. Nutr. Metab. Cardiovasc Dis. 35, 103787. 10.1016/j.numecd.2024.103787 39734133

[B41] Parish S. Arnold M. Clarke R. Du H. Wan E. Kurmi O. (2019). Assessment of the role of carotid atherosclerosis in the association between major cardiovascular risk factors and ischemic stroke subtypes. JAMA Netw. Open 2, e194873. 10.1001/jamanetworkopen.2019.4873 31150080 PMC6547114

[B42] Qiang Z. Wang Y. Li Y. Li Y. (2026). Association of the triglyceride-glucose index and phlegm-dampness constitution with subclinical carotid atherosclerosis in overweight and Obese individuals. Front. Cardiovasc. Med. 13, 1907694. 10.3389/fcvm.2026.1907694 PMC13481244 42614192

[B43] Roghani-Shahraki H. Karimian M. Valipour S. Behjati M. Arefnezhad R. Mousavi A. (2021). Herbal therapy as a promising approach for regulation on lipid profiles: a review of molecular aspects. J. Cell Physiol. 236, 5533–5546. 10.1002/jcp.30282 33469926

[B44] Shen D. Z. Zhao H. B. Xing S. L. Lou D. F. Shen R. Liu T. ,(2012). The preliminary evaluation of the clinical curative effect on carotid atherosclerosis patients for shoushen granules. Chin. J. Emerg. Traditional Chin. Med, 21(10):1559–1561.

[B45] Shen X. Zou S. Jin J. Liu Y. Wu J. Qu L. (2022). Dengzhan shengmai capsule versus aspirin in the treatment of carotid atherosclerotic plaque: a single-centre, non-inferiority, prospective, randomised controlled trial. Phytomedicine 106, 154408. 10.1016/j.phymed.2022.154408 36029646

[B46] Sizar O. Khare S. Patel P. Talati R. (2024). “Statin medications,”. *StatPearls*. Treasure Island, FL: StatPearls Publishing. 28613690

[B47] Song T. Chen W. D. (2021). Berberine inhibited carotid atherosclerosis through PI3K/AKTmTOR signaling pathway. Bioengineered 12, 8135–8146. 10.1080/21655979.2021.1987130 PMC8806982 34592881

[B48] Song P. Fang Z. Wang H. Cai Y. Rahimi K. Zhu Y. (2020). Global and regional prevalence, burden, and risk factors for carotid atherosclerosis: a systematic review, meta-analysis, and modelling study. Lancet Glob. Health 8, e721–e729. 10.1016/S2214-109X(20)30117-0 32353319

[B49] Spence J. D. Eliasziw M. DiCicco M. Hackam D. G. Galil R. Lohmann T. (2002). Carotid plaque area: a tool for targeting and evaluating vascular preventive therapy. Stroke 33 (12), 2916–2922. 10.1161/01.str.0000042207.16156.b9 12468791

[B50] Sterne J. A. C. Savović J. Page M. J. Elbers R. G. Blencowe N. S. Boutron I. (2019). RoB 2: a revised tool for assessing risk of bias in randomised trials. BMJ 366, l4898. 10.1136/bmj.l4898 31462531

[B51] Sun M. Zhang Z. Xie J. Yu J. Xiong S. Xiang F. (2024). Research progress on the mechanism for improving glucose and lipid metabolism disorders using phenolic acid components from medicinal and edible homologous plants. Molecules 29 (20), 4790. 10.3390/molecules29204790 39459158 PMC11510019

[B52] Sun G. Wang Z. Gao D. Fei E. (2025). Study on the combination of soft pulse and blood-activating traditional Chinese medicine compound with rosuvastatin calcium in the treatment of carotid atherosclerosis with kidney deficiency and blood stasis syndrome. Tradit. Chin. Med. Res. 38, 60–64. (in Chinese).

[B53] Thompson P. D. Panza G. Zaleski A. Taylor B. (2016). Statin-associated side effects. J. Am. Coll. Cardiol. 67, 2395–2410. 10.1016/j.jacc.2016.02.071 27199064

[B54] Touboul P. J. Hennerici M. G. Meairs S. Adams H. Amarenco P. Bornstein N. (2012). Mannheim carotid intima-media thickness and plaque consensus (2004–2006–2011): an update on behalf of the advisory board of the 3rd, 4th and 5th watching the risk symposia, at the 13th, 15th and 20th european stroke conferences, mannheim, Germany, 2004, brussels, Belgium, 2006, and Hamburg, Germany, 2011. Cerebrovasc. Dis. 34, 290–296. 10.1159/000343145 23128470 PMC3760791

[B55] Wang K. X. Zhang S. Q. (2024). Based on data mining to explore the medication rules of traditional Chinese medicine in the treatment of carotid atherosclerotic plaque. Asia Pac. Tradit. Med. 20, 190–195. (in Chinese). 10.11954/ytctyy.202410044

[B56] Wang P. J. Ai Y. H. Wei Y. C. Zhang X. Q. , (2014). The clinical research of Gegen Qinlian decoction on carotid artery atherosclerosis[J]. Med. Innovation China, 11(28): 19–21. 10.3969/j.issn.1674-4985.2014.28.007

[B57] Wei Y. L. Li Z. Q. Sun W. H. Huang Q. E. Tang Y. P. (2026). Xiaozhi huaban Fang combined with atorvastatin calcium tablets in the treatment of carotid atherosclerotic plaque with phlegm-turbidity and blood-stasis syndrome: a clinical study of 30 cases. J. Tradit. Chin. Med. 40 (10), 1067–1073. 10.19844/j.cnki.1672-397X.2024.10.009

[B58] Wu S. X. Wu X. L. Huang Z. X. Sun X. W. Lin Q. C. Lin B. H. (2000). Jiedu ruanmai Fang in anti-atherosclerosis: a preliminary clinical observation of 17 cases. Fujian J. Tradit. Chin. Med. 31 (5), 8–10. 10.13260/j.cnki.jfjtcm.2000.05.004

[B59] Wu T. M. Chen J. S. Du J. ,(2010). Clinical observation of kangxin capsule on carotid atherosclerosis with kidney deficiency and blood stasis syndrome[J]. Guangming J. Chin. Med, 25(8): 4382–4384. 10.3969/j.issn.1003-8914.2010.08.034

[B60] Xie J. B. Li W. B. Cao R. F. (2025). Efficacy and safety of modified banxia Baizhu tianma decoction in the treatment of hypertension complicated with carotid atherosclerotic plaque. Chin. J. Clin. Ration. Drug Use 17, 43–47. 10.15887/j.cnki.13-1389/r.2025.17.011

[B61] Yao K. W. (2026). Several key issues in prevention and treatment of carotid artery atherosclerotic plaques with traditional Chinese medicine. Acta Chin. Med. 41, 1–6. (in Chinese). 10.16368/j.issn.1674-8999.2026.01.001

[B62] Yuan Y. Yu H. Huangfu H. Liu Y. Liu C. (2025). Research progress on anti-atherosclerotic effects of Chinese medicine compound formulas based on the interaction mechanism between exosomes and cellular senescence. J. Hunan Univ. Chin. Med. 45, 2225–2231. (in Chinese). 10.3969/j.issn.1674-070X.2025.11.029

[B63] Zeng J. Cai H. R. Zhao S. Li Z. S. Zhang W. Z. Zhang L. Y. (2021). Analysis of curative effect of shugan wendan decoction in the treatment of atherosclerosis. Mod. J. Integr. Tradit. Chin. West Med. 30 (26), 2856–2860. 10.3969/j.issn.1008-8849.2021.26.002

[B64] Zhang Y. H. , Clinical Study of Xiaoban Tongmai Prescription on 120 Cases of Carotid plaque[C]//Proceedings of the Third China Traditional Chinese Medicine Science and Technology Achievement Forum. Place unknown: Publisher unknown, Year unknown: Page unknown.

[B65] Zhang M. Liu Y. Xu M. Zhang L. Liu Y. Liu X. (2019). Carotid artery plaque intervention with tongxinluo capsule (CAPITAL): a multicenter randomized double-blind parallel-group placebo-controlled study. Sci. Rep. 9, 4545. 10.1038/s41598-019-41118-z 30872737 PMC6418108

[B66] Zhang S. Hong F. Ma C. Yang S. (2022). Hepatic lipid metabolism disorder and atherosclerosis. Endocr. Metab. Immune Disord. Drug Targets 22, 590–600. 10.2174/1871530322666211220110810 34931971

[B67] Zhang F. Xu Y. Y. Shen L. Y. Huang J. J. Xu S. T. Chen M. L. (2023). Pathological mechanism of “Phlegm, Blood Stasis, Toxin” in a rabbit model of carotid atherosclerosis based on gut microbiota-Host metabolism interactions. Clin. Complementary Med. Pharmacol. 3 (2), 100056. 10.1016/j.ccmp.2022.100056

[B68] Zhao R. N. Rong Z. Shao J. (2023). Clinical efficacy of xiaorong wenban formula on unstable carotid atherosclerotic plaques in patients with qi deficiency and phlegm-stasis blockage syndrome. Lishizhen Med. Materia Medica Res. 34 (11), 2701–2705. 10.3969/j.issn.1008-0805.2023.11.33

[B69] Zhong G. S. Yang B. C. Chinese Materia Medica (2021). New Century. 5th Edition. Beijing: China Press of Traditional Chinese Medicine.

[B70] Zhong M. Y. Wang C. Y. Zhuang M. An J. L. Du X. C. Zhai J. N. (2023). Problems and thoughts in clinical safety evaluation of traditional Chinese medicine. China J. Chin. Materia Med 48, 3404–3408. (in Chinese). 10.19540/j.cnki.cjcmm.20230123.503 37382023

[B71] Zou T. Wang J. Wu X. Yang K. Zhang Q. Wang C. L. (2023). A review of the research progress on Pinellia ternata (thunb.) breit.: botany, traditional uses, phytochemistry, pharmacology, toxicity and quality control. Heliyon 9 (12), e22153. 10.1016/j.heliyon.2023.e22153 38058630 PMC10696046

